# Ex-TFRs: A Missing Piece of the SLE Puzzle?

**DOI:** 10.3389/fimmu.2021.662305

**Published:** 2021-04-09

**Authors:** Xundong Wei, Jianhua Zhang, Xuyu Zhou

**Affiliations:** ^1^ Center of Biotherapy, Beijing Hospital, National Center of Gerontology, Institute of Geriatric Medicine, Chinese Academy of Medical Sciences, Beijing, China; ^2^ Savaid Medical School, University of Chinese Academy of Sciences, Beijing, China; ^3^ CAS Key Laboratory of Pathogenic Microbiology and Immunology, Institute of Microbiology, Chinese Academy of Sciences (CAS), Beijing, China

**Keywords:** systemic lupus erythematosus, T regulatory cell, ex-TFRs, Foxp3, stability, autoantibodies

## Abstract

Systemic lupus erythematosus (SLE) is a chronic multi-organ autoimmune disease involving the production of a wide range of autoantibodies and complement activation. The production of these high-affinity autoantibodies requires T cell/B cell collaboration as well as germinal center (GC) formation. T follicular regulatory cells (TFRs) are functional specialized T regulatory cells (Tregs) that safeguard against both self-reactive T and B cells. However, recent evidence suggests that TFRs are not always stable and can lose Foxp3 expression to become pathogenic “ex-TFRs” that gain potent effector functions. In this review, we summarize the literature on intrinsic and extrinsic mechanisms of regulation of TFR stability and discuss the potential role of TFR reprogramming in autoantibody production and SLE pathogenesis.

## Introduction

Systemic lupus erythematosus (SLE) is a chronic multi-organ autoimmune disease with wide clinical heterogeneity. The disease can cause injury to many organs, especially the kidneys, joints, and skin. The pathogenesis of SLE is not fully understood, but increased high-affinity self-antibody production and dysregulated immune tolerance have been implicated in the progression of the disease (1, 2). SLE often starts in late childhood or adolescence and predominantly affects females in their reproductive years, with a female/male ratio of 9:1; the reasons for this skewed sex ratio remain unclear (3, 4). In the 1950s, only 50% of SLE patients survived for 5 years; now, due to early diagnosis and better treatment, most patients survive for more than 10 years. There is no effective treatment for SLE, and only a few drugs have been approved in the past 60 years, emphasizing the need for a better understanding of its pathogenesis.

A hallmark of SLE is the production of a wide range of autoantibodies by self-reactive B cells. Anti-nuclear antibodies (ANAs) are detected in >95% of SLE patients, and subsequent deposition of immune complexes in endogenous tissue results in severe tissue damage and induction of inflammation (5, 6). The autoantibodies from lupus patients are high affinity, somatic mutated, and class switched, and their generation requires the formation of germinal centers (GCs) with assistance from follicular helper T cells (TFHs) (7). In addition to their involvement in GC formation, TFHs, a unique CD4^+^ subset of T cells with high expression of Bcl6, PD-1, and CXCR5, play a major role in the selection of high-affinity B cells. TFHs have thus emerged as a critical immunoregulator of antibody production as well as the pathogenesis of human SLE (8).

Another small population of CD4^+^ T cells, regulatory T cells (Tregs), maintain self-tolerance by suppressing both autoreactive T and B lymphocytes through the production of inhibitory cytokines such as IL-10, TGF-β, and IL-35 (9, 10). Similar to conventional T cells, TCR and MHC and peptide engagement will lead to the activation of Tregs and which contribute to the further development of functional specialized T follicular regulatory cells (TFRs). TFRs also express both Bcl6 and CXCR5 and are capable of traveling to B cell follicles to serve as gatekeepers controlling autoantibody production (11–13). Tregs were initially considered a stable lineage, but emerging evidence suggests that even fully committed Treg cells can lose their identity and be reprogrammed to effector T cells (14–17). Interestingly, reprogramming of Tregs has been observed in the follicular region. TFRs can lose their Foxp3 expression and become pathogenic “ex-TFRs” (18). Whether the autoreactive potential of ex-TFRs contributes to autoimmune disease is not known. In this review, we summarize recent progress in understanding the roles of signaling pathways and transcriptional and epigenetic regulation in modulating Treg and TFR stability. We also discuss the possibility that pathogenic ex-TFRs contribute to autoantibody production and the pathogenesis of SLE.

## TFRs

In 1995, Sakaguchi et al. identified a small subset of CD4^+^ T cells that express the high-affinity IL-2 receptor IL-2Rα (CD25) and are capable of suppressing autoimmunity upon transfer (19, 20). Indeed, mice lacking either IL-2Rα or IL-2 develop severe systemic autoimmunity (21–23). The cells identified by Sakaguchi et al. are now known as Tregs, and in 2003, the transcription factor Forkhead Box P3 (Foxp3) was identified as the lineage-defining regulator of Tregs (24–26). The importance of Foxp3 has been illustrated by studies of Foxp3 gene mutations, immune dysregulation, polyendocrinopathy, enteropathy, and X-linked (IPEX) syndrome in humans, and Scurfy mutant mice bearing Foxp3 mutations develop lethal multi-organ autoimmunity (27–30). In addition, ablation of Foxp3 in mature Tregs or depletion of Foxp3^+^ cells completely eliminates the suppressive capacity of Tregs and programs Tregs into pathogenic T cells (31).

Tregs are not a homogenous population. Depending on their developmental origin, Tregs can be divided into thymic Tregs (tTregs) and peripheral Tregs (pTregs) (32, 33). tTregs are induced in the thymus and are characterized by high-affinity self-antigen engagement (34). By contrast, pTregs are generated from conventional CD4^+^ T cells under conditions of high levels of transforming growth factor β (TGF-β) and retinoic acid in the environment or in response to metabolites produced by microbiota in the gut (35). Although the TCR repertoires of pTregs and tTregs overlap, tTregs mainly recognize self-antigens, whereas pTregs also express TCRs specific for non–self-infectious antigens or innocuous commensal microbiota derived antigens; these latter TCRs are important for the maintenance of mucosal tolerance (36, 37).

Similar to conventional T cells, Treg TCR engagement is the first step in generating heterogeneous effector Tregs, which are functionally potent and capable of migrating to local tissue (38). Effector Tregs can differentiate into specialized subsets by adapting the same set of transcription factors that control the differentiation of helper CD4^+^ T cells. For example, the Th1 transcription factor T-bet drives the expression of CXCR3 in Tregs, which is important for regulating some Th1-mediated autoimmune responses (39), and the RORγt-expressing Treg is involved in the regulation of Th17-mediated experimental autoimmune encephalomyelitis (EAE) and colitis (40–42).

Bcl6-expressing TFR cells (TFRs) are a particularly important subset of effector Tregs that express CXCR5 and migrate to B cell follicles and GCs (11). TFRs are capable of modulating B cell responses and, given their unique localization, appear to be major players in controlling autoantibody production (43–48). Indeed, Treg-specific ablation of Bcl6 results in substantial increases in multiple autoreactive antibodies, including anti-dsDNA (49, 50). Sage et al. demonstrated the importance of TFR in controlling the production of a panel of self-antibodies in an elegant TFR-DTR model established by crossing Foxp3-Cre mouse with a CXCR5 floxed stop DTR (diphtheria toxin receptor) strain (12, 51). In this model, only cells expressing both Foxp3 and CXCR5 (such as TFRs) express DTR on the cell surface, making them susceptible to deletion upon administration of diphtheria toxin (DT).

TFRs differentiate from natural Treg precursors through interaction with dendritic cells (DCs) and require different costimulatory activation signals at different stages of differentiation. Treg cells do not express CXCR5 in the T-cell zone and only start to upregulate CXCR5 when they migrate to the border region between the T and B cell zones. These cells are defined as pre-TFRs (52). The early differentiation of pre-TFRs requires CD28 and ICOS helper signals from DC cells and is independent of B cells. Although the initial stage of TFR differentiation does not rely on B cells, the stable mature TFR program requires B cell assistance. B cell-deficient mice exhibit a large decrease in mature TFRs in the lymph nodes (47). In follicles, CD25 and Blimp-1 expression are downregulated in CD25^+^ TFRs, leading to the acquisition of the CXCR5^hi^Bcl6^hi^ phenotype, which allows these CD25^-^ TFRs to traffic into the GC (**Table 1**). Sage and colleagues demonstrated that TFRs prevent self-reactive B cells from being activated by TFHs, most likely *via* attenuated production of cytokines (such as IL-21 and IL-4) and/or costimulatory signals (53, 54). TFRs also prevent GC formation caused by foreign antigens (vaccines, microorganisms) by inhibiting the metabolic flux of B cells and through CTLA-4-mediated inhibition of B cells. TFRs may physically interrupt bidirectional costimulation and linked recognition during immunological synapses between TFHs and B cells (55). A specific subtype of TFHs, SOSTDC1^+^ TFHs, promote TFR cell differentiation by inhibiting the β-catenin pathway through the secreted protein SOSTDC1 (56).

**Table 1:** The markers for the identification of TFR and TFH.

Subtype	Foxp3	CD25	Blimp-1	CXCR5	PD-1	ICOS	Bcl6	CD44	CD62L	Location	Autoreactive
**Naive Treg**	+	+	−	−	−	−	−	−	+	Extrafollicle	High
**Pre-TFR**	++	++	+	+	+	+	+/-	+	–	T-B border	High
**CD25^+^TFR**	++	++	++	++	++	++	++	+	−	Follicule	High
**CD25^-^TFR**	++	−	−	+++	+++	+++	+++	+	−	GC	High
**Ex-TFR**	−	−	−	+++	+++	+++	+++	+	−	GC	High
**Naive T**	−	−	−	−	−	−	−	−	+	Extrafollicle	Low
**Pre-TFH**	−	−	−	+	+	+	+/-	+	–	T-B border	Low
**TFH**	−	−	−	++	++	++	++	+	–	Follicule	Low
**GC-TFH**	−	−	−	+++	+++	+++	+++	+	–	GC	Low

Defects in Treg function and/or number, particularly the TFR subset, are thought to contribute to SLE pathogenesis, but conflicting results have been reported. Some groups have found an increase in TFRs in SLE patients compared with healthy controls (57–59), whereas others have found reduced numbers or impaired function of circulating TFRs or Tregs in SLE patients (60, 61). Other groups have observed no abnormalities (62, 63). These discrepancies are due in part to the lack of a unique marker or combination of markers for identifying and isolating bona fide Tregs, the use of different *in vitro* stimuli, and the presence or absence of antigen-presenting cells (APCs) in *ex vivo* functional assays (64). An important challenge in the study of the pathogenesis of SLE is the difficulty of obtaining patient lymphoid tissues to assess TFRs directly; for this reason, most studies have focused on circulating Tregs in peripheral blood.

## Foxp3 Stability of TFRs

Tregs were initially considered a stable cell lineage committed to immunosuppressive function, but accumulating evidence indicates that they can lose Foxp3 expression and undergo reprogramming to other types of effector T cells. Upon transfer into CD3e KO mice, Foxp3^+^CD4^+^ T cells terminate Foxp3 expression and differentiate into TFHs in Peyer’s patches (14). CD25^-^Foxp3^+^ T cells are likely more unstable than cells expressing CD25. In a fate-mapping experiment involving Foxp3 bacterial artificial chromosome (BAC) transgenic mice expressing GFP-Cre under the control of the Foxp3 promoter, we demonstrated that a fraction of Tregs are not stable. These “ex-Tregs”, which no longer express Foxp3, have an activated-memory T cell phenotype and the ability to produce inflammatory cytokines such as IFN-γ and IL-17. Importantly, ex-Tregs bearing the BDC2.5 TCR induce autoimmune diabetes upon adoptive transfer (15). Autoimmune inflammation exacerbates the instability of Foxp3. By using MOG tetramer to identify antigen-specific Tregs, we further demonstrated that Tregs can be converted into pathogenic T helper cells in an EAE mouse model, suggesting a link between strong TCR signaling and Treg instability (16).

As a major TGF-β sensor, conserved noncoding sequence 1 (CNS1) in Foxp3 is critical for the generation of induced pTregs but dispensable for tTreg development. Given the heterogeneity of Tregs, we further generated a delta CNS1 Foxp3 BAC transgenic mouse strain that only traces committed and stable tTregs (17). We found that resting or naïve tTregs are stable, but upon development to TFRs, these cells can lose Foxp3 stability and be reprogrammed into a T helper lineage (17).

Sage et al. recently confirmed that a population of TFRs can lose Foxp3 expression in experiments using inducible Foxp3 fate-mapper mice (FoxP3^ERT2-Cre^-Rosa26 Lox-Stop-Lox-TdTomato). In this model, Cre–ERT2 is limited to the cytoplasm in the absence of tamoxifen. Upon administration of tamoxifen, the tamoxifen metabolite 4-OHT (an analog of estrogen) binds to ERT, allowing Cre-ERT2 to enter the nucleus and exert Cre recombinase activity, thus triggering the expression of the fluorescent protein TdTomato in Foxp3^+^Treg cells. In contrast to continuous labeling, inducible labeling of Foxp3^+^Tregs with TdTomato during the immunization period avoids cell labeling due to transient Foxp3 expression and permits the assessment of bona fide Treg maintenance. Sage et al. immunized these mice with NP-OVA and 7 d later assessed the frequency of CXCR5^+^CD4^+^TdTomato^+^Foxp3^low^ “ex-TFR” populations. In this model, ∽80% of CD4^+^CXCR5^+^TdTomato^+^ cells retained Foxp3 (TFRs); the remaining ∽20% lost Foxp3 expression (ex-TFRs).

**Figure 1:**
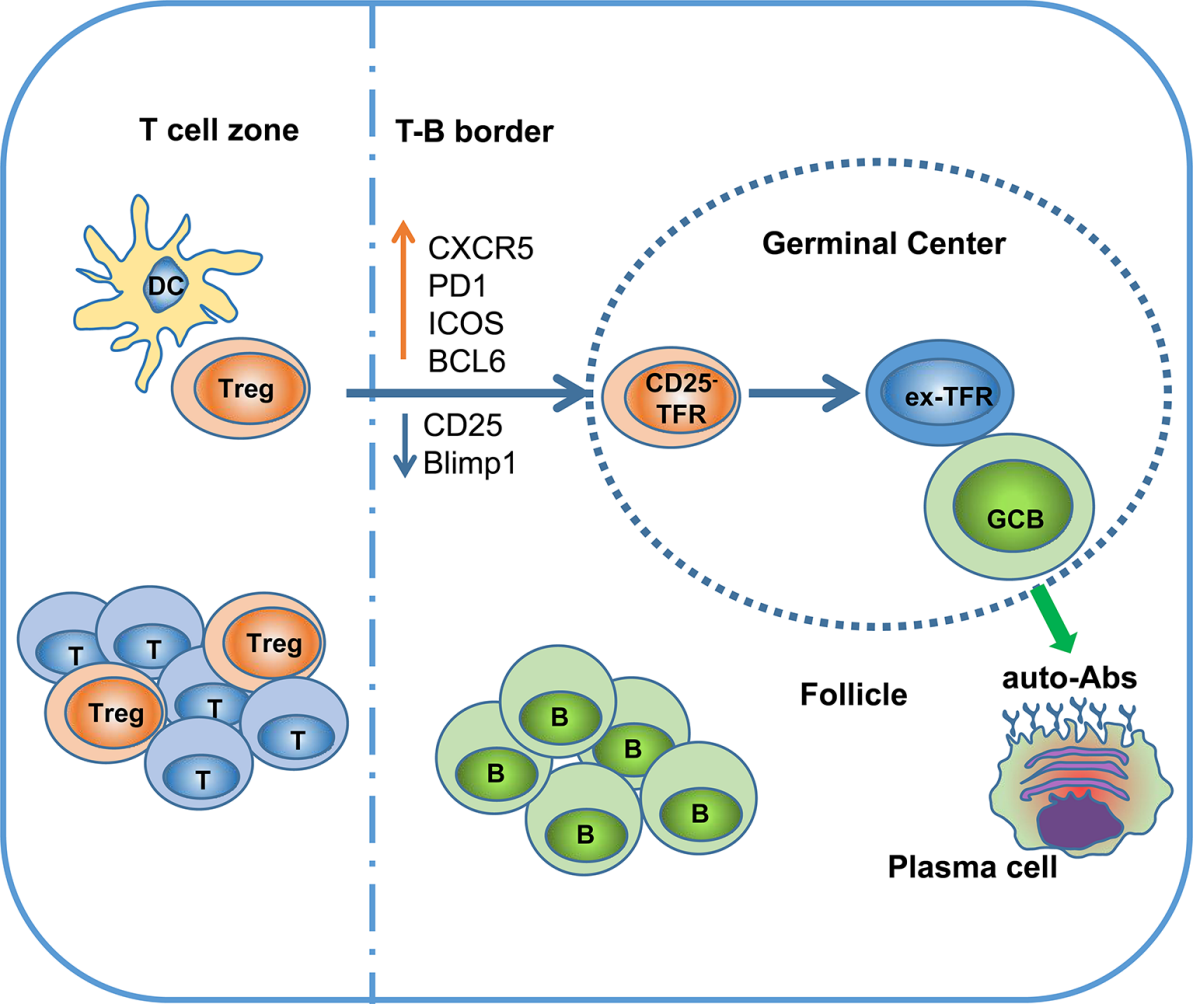
Ex-TFRs: a missing piece of the SLE puzzle. Naïve Treg cells can interact with dendritic cells (DCs) to become activated and further migrate into the germinal center (GC) region through upregulation of CXCR5 and BCL6. In the follicle and GC, TFRs play an important role in regulating antigen (Ag)-specific TFHs and antibody-secreting cells. Since the GC is not favorable to stable Foxp3 expression, some TFRs will lose Foxp3 and develop into pathogenic TFHs (ex-TFRs). Ex-TFRs tend to recognize self-antigens, which may promote autoreactive humoral immunity.

In summary, although TFRs are a functionally specialized subset of Tregs that selectively survey the autoreactive antibody response in the GC, continuous localization of TFRs in the GC might have a detrimental effect on Treg stability, leading to loss of Foxp3 expression and reprogramming to TFHs (**Figure 1**) (18).

## Why Do TFRs Preferentially Lose Foxp3?

The stability of Foxp3 expression is largely determined by the methylation status of the CNS2 region of the Foxp3 gene locus, which is also known as the Treg-specific demethylated region (TSDR) (65, 66). Foxp3 CNS2 contains 11 CpG sites, which are all methylated in peripheral conventional T cells as well as thymic DP and CD4 SP cells. Gradual demethylation of CNS2 occurs during tTreg development (67, 68); this process is not passively cell cycle dependent but is mediated by Tet-dependent oxidation, which is primarily mediated by Tet2 and Tet3 (69). Demethylation of CNS2 leads to the recruitment of transcription factors such as Cbfβ, Runx1, STAT5, and Foxp3 itself, thereby reinforcing Foxp3 expression on Tregs (65, 70–72). Indeed, genetically deleting either the CNS2 enhancer of the Foxp3 locus or Tet family proteins leads to a destabilized Treg lineage and the development of spontaneous autoimmunity and chronic inflammation (73).

Treg stability is influenced by many intrinsic and extrinsic factors, particularly cytokines and their downstream signaling pathways. IL-2 and STAT5 activation maintain Foxp3 stability by binding directly to CNS2, and the Hippo kinases Mst1 and Mst2 promote STAT5 activation to further strengthen the Treg lineage (74, 75). By contrast, IL-4 and IL-6 can have detrimental effects on the Treg lineage. IL-4 receptor (IL-4R) knock-in mice in which IL-4R signaling is specifically upregulated exhibit reduced Treg stability and promotion of the Th2 response (76). STAT6 and STAT3, which are downstream of IL-4R, appear to compete with STAT5 at the CNS2 region of Foxp3. Depletion of SOSC1 (Socs1^fl/fl^ × Foxp3^YFP-Cre^ mice), a natural inhibitor of STAT proteins, destabilizes the Treg lineage, and more interestingly, adoptive transfer of SOSC1-deficient Tregs is sufficient to induce autoimmune colitis (77).

Although ablation of TCR in mature Tregs has little impact on Treg stability, overstimulation of Tregs *via* dysregulation of TCR and/or co-stimulation profoundly destabilizes the Treg lineage. A number of negative regulators of the TCR signaling pathway, such as PTEN, ITCH, Vhl, and PTPN, play important roles in maintaining the stability of the Treg lineage (78–80). Interestingly, Tregs themselves are partially anergic compared with conventional T cells. Under conditions of homeostasis, Tregs remain anergic, but TCR signaling upon weak stimulation confers strong suppressive potential on Tregs without reducing the stability of the lineage (81, 82). However, overstimulation causes Treg destabilization and reprogramming into pathogenic effector cells (17, 80, 83). The detailed molecular mechanism of the TCR signaling pathway has not been fully elucidated, but metabolic mechanisms could be very important; some metabolic pathways may interact with transcriptional and epigenetic regulation to modulate the Treg lineage.

Another important regulator of Treg lineage maintenance is the Foxp3 complex itself; roles of EzH2, RelA, and Runx, among other components of the complex, have been demonstrated (84, 85). Post-translational modification of the Foxp3 protein is part of a feedback loop that controls Foxp3 stability. CRISPR-Cas9-based screening is beginning to reveal a more comprehensive picture of Treg lineage regulation, and new regulators such as Usp22, Rnf20, and Brd9 have been identified (86, 87). Interestingly, Zheng et al. found that non-canonical BAF (ncBAF) can localize at Foxp3 cis-regulatory elements to promote Foxp3 binding, whereas another SWI/SNF subunit, PBAF, seems to exert opposing effects (88).

In addition to its critical role in maintaining Foxp3 stability (89, 90), the IL-2/STAT5 signaling pathway is a potent negative regulator of TFH differentiation. IL-2 has been reported to repress TFR differentiation by a STAT5/Blimp-1 dependent mechanism (91). Thus, downregulation of the high-affinity IL-2 receptor CD25 is likely a common strategy for avoiding excessive STAT5 signaling in TFRs and TFHs. Consistent with this notion, CD25 expression is typically low or absent on TFHs and TFRs (92). By contrast, TFRs express high levels of inducible costimulator (ICOS), a co-stimulation molecule belonging to the CD28 family. ICOS signaling through the PI3K/AKT pathway is essential for the initiation of TFH and TFR differentiation and is also an important survival signal for CD25^-^ effector Tregs (93–95). However, as mentioned above, such positive signals can dampen Treg stability; for example, loss of Blimp-1, a strong transcriptional repressor of Bcl6, boosts TFR differentiation but has a detrimental effect on Treg stability (96). Together, the CD25 and ICOS signal switch during TFR cell differentiation is the driving force for programming TFRs to become TFHs (96).This notion is also consistent with previous adoptive transfer experiments showing that CD25^-^Foxp3^+^ cells preferentially differentiate into effector TFHs under lymphopenic conditions (**Table 1**).

## Ex-TFRs: A Missing Piece of the SLE Puzzle?

The functional role of ex-TFRs is not fully understood. The large majority of TFRs express Helios, a transcription factor expressed by tTregs (97), suggesting that TFRs are thymic in origin and biased toward self-antigens. This notion is further supported by recent studies indicating a highly diverse TCR repertoire of TFRs (36, 37). The loss of Foxp3 expression on TFRs generates a population of T cells with the potential to attack self-tissue. These cells could have a similar function as autoreactive TFHs. Sage et al. showed that ex-TFRs lose their suppressive function and have a transcriptional signature that is more similar to TFHs than TFRs (18). Moreover, multiple lines of Treg conditional knockout mice exhibit defects in maintenance of Treg stability, in association with an increased autoreactive humoral response and even the development of lupus-like autoimmune disorders (98–100). For example, mice in which PTEN is conditionally knocked out in Tregs develop a lupus-like autoimmune lymphoproliferative disease characterized by excessive levels of TFHs and B cell activation. These mice also exhibit increased serum levels of multiple auto-antibodies and creatinine, indicating renal pathology (80, 101). Tet2/3 conditional knockout mice develop lethal autoimmunity in association with the production of numerous self-antibodies (73), and a similar autoimmune disease is observed in Foxp3Cre^WT/Cre^Tet2/3^fl/fl^ heterozygous female mice, which harbor half of the wild type of Tregs in the same mice (73). These results strongly support the notion that ex-Tregs are self-recognition biased and have pathogenic potential (**Figure 1**).

Treg stability has not been directly tested in mouse models of lupus or human patients. However, Benoist et al. found that Foxp3^+^ Treg cells are unstable in NZW mice, which may explain the reduced sensitivity of this NZW Tregs to limiting doses of trophic cytokines, IL-2 and -33 (102). In addition, this instability may provide a genetic explanation for disease pathogenesis, as NZW × NZB F1 female mice develop a severe autoimmune disease that shares many features of SLE in human patients (103).

## Conclusions

SLE is an autoimmune disease characterized by the production of a wide range of autoantibodies, and its exact pathoetiology remains elusive. Although TFRs play a critical role in controlling autoantibody production, the migration of TFRs to the follicular region and GC does not favor stable Foxp3 expression, and some TFRs even lose Foxp3 and develop into TFHs with pathogenic potential. These ex-TFRs are likely biased toward self-recognition and might promote autoreactive humoral immunity. A better understanding of the role of ex-TFRs could have important therapeutic implications for SLE and many other autoimmune diseases.

## Author Contributions

XW, JZ, and XZ wrote the manuscript. All authors contributed to the article and approved the submitted version.

## Funding

This work was supported by the National Natural Science Foundation of China (31870911, 81802872), the National Science and Technology Major Project of China (2016ZX10004222-007 and 2018ZX10301-208-002-002).

## Conflict of Interest

XW consulted for Zhongke Qiyuan (Shenzhen) Biotechnology Co., Ltd. in T cell immunity.

The remaining authors declare that the research was conducted in the absence of any commercial or financial relationships that could be construed as a potential conflict of interest.
